# The effect of gender on the neuroanatomy of children with autism spectrum disorders: a support vector machine case-control study

**DOI:** 10.1186/s13229-015-0067-3

**Published:** 2016-01-19

**Authors:** Alessandra Retico, Alessia Giuliano, Raffaella Tancredi, Angela Cosenza, Fabio Apicella, Antonio Narzisi, Laura Biagi, Michela Tosetti, Filippo Muratori, Sara Calderoni

**Affiliations:** Istituto Nazionale di Fisica Nucleare, Pisa Division, Largo B. Pontecorvo 3, 56127 Pisa, Italy; University of Pisa, Department of Physics, Largo B. Pontecorvo 3, 56127 Pisa, Italy; IRCCS Stella Maris Foundation, viale del Tirreno 331, 56018 Pisa, Italy; University of Pisa, Department of Clinical and Experimental Medicine, Via Savi 10, 56126 Pisa, Italy

**Keywords:** Autism spectrum disorders, Structural MRI, Support vector machine, Young children, Gender differences

## Abstract

**Background:**

Genetic, hormonal, and environmental factors contribute since infancy to sexual dimorphism in regional brain structures of subjects with typical development. However, the neuroanatomical differences between male and female children with autism spectrum disorders (ASD) are an intriguing and still poorly investigated issue. This study aims to evaluate whether the brain of young children with ASD exhibits sex-related structural differences and if a correlation exists between clinical ASD features and neuroanatomical underpinnings.

**Methods:**

A total of 152 structural MRI scans were analysed. Specifically, 76 young children with ASD (38 males and 38 females; 2–7 years of age; mean = 53 months, standard deviation = 17 months) were evaluated employing a support vector machine (SVM)-based analysis of the grey matter (GM). Group comparisons consisted of 76 age-, gender- and non-verbal-intelligence quotient-matched children with typical development or idiopathic developmental delay without autism.

**Results:**

For both genders combined, SVM showed a significantly increased GM volume in young children with ASD with respect to control subjects, predominantly in the bilateral superior frontal gyrus (Brodmann area –BA– 10), bilateral precuneus (BA 31), bilateral superior temporal gyrus (BA 20/22), whereas less GM in patients with ASD was found in right inferior temporal gyrus (BA 37). For the within gender comparisons (i.e., females with ASD vs. controls and males with ASD vs. controls), two overlapping regions in bilateral precuneus (BA 31) and left superior frontal gyrus (BA 9/10) were detected. Sex-by-group analyses revealed in males with ASD compared to matched controls two male-specific regions of increased GM volume (left middle occipital gyrus—BA 19—and right superior temporal gyrus—BA 22). Comparisons in females with and without ASD demonstrated increased GM volumes predominantly in the bilateral frontal regions. Additional regions of significantly increased GM volume in the right anterior cingulate cortex (BA 32) and right cerebellum were typical only of females with ASD.

**Conclusions:**

Despite the specific behavioural correlates of sex-dimorphism in ASD, brain morphology as yet remains unclear and requires future dedicated investigations. This study provides evidence of structural brain gender differences in young children with ASD that possibly contribute to the different phenotypic disease manifestations in males and females.

**Electronic supplementary material:**

The online version of this article (doi:10.1186/s13229-015-0067-3) contains supplementary material, which is available to authorized users.

## Background

One of the most evident distinctions between males and females with autism spectrum disorder (ASD) regards the well-documented male preponderance ranging from approximately 1.33:1 to 16.0:1, depending on sites, subtypes of ASD diagnosis and intelligence quotient (IQ) level [1]. The etiopathogenesis of this gender bias remains a matter of thoughtful debate [2]; while some authors question the significance of male preponderance and ascribe it to a greater under-diagnosis or wrong diagnosis of females with ASD [3], others trace back the biased sex ratio to a genetic [4] and/or sex-related hormones pathogenesis [5]. Nevertheless, the male-to-female differences in patients with ASD extend beyond the epidemiological perspective to include clinical and genetic/neurobiological aspects [6]. In fact, the literature suggests that the clinical presentation of individuals on the autism spectrum may vary with respect to gender (see [7] for a very large dataset of sex differences in behavioural data of adults with ASD and [8] for a review). Restricting the analysis to childhood studies, older female children with ASD seem to exhibit a more severe phenotype, in terms of lower IQ [9], impairment in adaptive functioning [10], autistic symptoms [11] and psychopathological problems [12]. In addition, females with ASD present worse performances on tests of global executive functions and theory of mind [13], as well as on the Block Design subtest of WISC-III [14]. However, data on sex differences in behavioural manifestations and adaptive functioning of children with ASD are still limited and inconclusive. First of all, a difficulty in identifying females with ASD is reported; these patients are diagnosed later in life than males, especially if coexistent intellectual or behavioural problems are present [15, 16]. The few studies that have explored this issue indicate a better performance of young males on communicative and fine-motor skills, while females show better visual receptive abilities [17, 18]. Moreover, males exhibit more restricted, repetitive, stereotyped behaviour than females [18, 19], and females more comorbid psychopathology [18]. More recently, a large investigation on 2418 probands with ASD concluded that females have a different cognitive and behavioural profile than males [20], characterized by a higher prevalence of intellectual disability that negatively impact the level of socio-communicative skills and adaptive function. On the other hand, other studies reported no significant gender differences in the symptom profile of preschoolers with ASD [21] or gender differences that reflect those found in typical young children [22]. In a similar vein, a systematic review and meta-analysis of the impact of age and gender on the core autistic symptoms described no differences in symptom severity between toddler/preschooler males and females with ASD [23].

Only a few studies have examined if and how behavioural differences between males and females with ASD mirror at the neuroanatomical level. Previous structural magnetic resonance imaging (sMRI) studies focused on young males and females with ASD independently frequently described different neuroanatomical profiles between genders. For example, an enlargement of total cerebral volume distinguished males, but not females with regressive autism from subjects with typical development [24]. Conversely, Sparks et al. [25] found similar enlargement of cerebral volume across gender in ASD young children, but the sample of females with ASD was limited to seven subjects. When sex differences in regional brain anatomy are taken into account, an enlargement of the amygdala volume more evident in females with ASD than males was detected [26]. In addition, an enlargement of the temporal regions and a reduction in cerebellar grey matter volume was typical of females with ASD only [27]. Finally, a longitudinal study that follows patients with ASD through early childhood evidenced a more widespread and severe brain growth trajectory in females than in males [28]. However, until a recent paper on 22 female children with ASD [24], whole brain sMRI studies on young female ASD were restricted to relatively small samples sizes of fewer than ten females [25–28], which limited their reliability [29].

Thus, a preponderance of evidence clearly indicates various and often unreplicated cerebral differences between sex in patients with ASD, with females generally showing more pronounced cerebral alteration as compared to males. However, methodological limitations, such as small sample sizes, different ASD phenotypes and variability of analysis techniques, hamper a univocal interpretation of findings.

Differences in brain developmental trajectories between individuals with ASD and typical subjects should also be mentioned. During the toddler and early childhood years of typical development, the brain undergoes a rapid dynamic phase of dendritic arborization, synaptic pruning and axon myelination. This process is reflected in a dramatic grey matter (GM) growth in the first few years of life followed by GM volume decrease after 4–5 years of age, and in white matter (WM) volume increase, with age reaching a plateau in late adolescence [30–32]. Conversely, the brains of at least a subgroup of individuals with ASD exhibit early overgrowth of WM and GM before 2 years of age [28, 33, 34] followed by a premature decrease of growth rate that lead to brain volumes more closely matched to typical children by approximately the school-age period [35, 36].

In this study we evaluated the effect of gender on the brain structure of patients with ASD using the support vector machine (SVM) analytic approach [37]. SVM is increasingly spreading machine-learning techniques suitable to extract information from MRI data in order to carry out single-subject classification of pathological states or prediction of pathology progression [38]. The introduction of pattern classification and computer vision methods in the neuroimaging field is due to Lao et al. [39]. With the aim to develop an accurate predictor of pathology from a set of volumetric images the authors highlighted the limitations of voxel-based morphometry methods (VBM) [40] and the potential of SVM-based decisional systems. Since then, SVM classifiers have been applied to investigate on a large variety of pathologies, involving the analysis of different types of brain data, e.g. sMRI [41–45], functional MRI (fMRI) [46], diffusion tensor imaging (DTI) [47], as well as features extracted from segmented brain regions [48], or combination of features extracted from different diagnostic modalities [49]. Some scepticism accompanies this spreading use of decision-making systems, especially when they are applied to support the diagnosis of psychiatric illness [50]. Practical difficulties in that case have still to be addressed before the SVM single-subject classification ability demonstrates its diagnostic utility and moved into the clinic. The studies reporting on SVM multivariate techniques applied to brain sMRI data of patients with ASD (for a recent review see [51]) support an increasing interest in the application of pattern classification methods as they provide inferences at single-subject level, and they allow the detection of subtle interesting abnormal patterns unobservable by univariate methods [41, 45]. With particular interest in the latter characteristic of the SVM approach, we deepened the investigation of the ASD pathology by implementing the SVM analysis of the sMRI data of a group of patients with ASD and control subjects.

In the present paper, we compared a large sample of male and female young children with ASD to controls carefully matched for age-, gender- and non-verbal-IQ (NVIQ). In particular, the aims of this study are to test: (1) neuroanatomical differences between young children with ASD and matched controls using SVM; (2) cerebral structural differences between genders in ASD patients with ASD detected by SVM; and (3) associations between ASD clinical symptomatology and neuroanatomical correlates.

## Methods

### Participants

A total of 152 subjects were analysed for this study: 76 patients with ASD, including 38 males in the 27–87-month range and 38 females in the 25–88-month range; 76 controls, including 38 males in the 24–88-month range and 38 females in the 22–89-month range. The inclusion and exclusion criteria for patients with ASD and controls were extensively described elsewhere [44] and were reported in the Additional file 1. Within the group of patients with ASD, we analysed 38 males and 38 females who were matched one-by-one for chronological age, and NVIQ. These pairs were selected from a total sample of 214 patients who met Diagnostic and Statistical Manual of Mental Disorders, 4th Edition Text Revision (DSM-IV-TR), criteria for a diagnosis in the autism spectrum and underwent a MRI scan. They were either in-patients or day patients at the ASD Unit of IRCCS Stella Maris Foundation, a tertiary hospital and research university. The control group was composed of 38 children (19 males and 19 females) with idiopathic developmental delay (DD), i.e. with NVIQ score <70, and 38 children without DD (19 males and 19 females), i.e. with NVIQ score ≥70. Similar to other studies [34, 52], we included subjects with DD within the control group to obtain a match for NVIQ between patients and controls. The control group of subjects with DD was very accurately selected (see Additional file 1), including the specific requirements of no family history of ASD, of a score below 20 on the Childhood Autism Rating Scale (CARS), and of the absence of any suspicions of an ASD by experienced clinicians’ judgement. Moreover, the sample of control subjects with DD does not satisfy the diagnostic criteria for Social (Pragmatic) Communication Disorder and Stereotypic Movement Disorder, according to the DSM-5.

Patients with ASD (*n* = 76), controls with DD (*n* = 38) and controls without DD and borderline intellectual functioning (NVIQ between 70 and 85) (*n* = 20) underwent the brain MRI examination as a completion of the assessment pathway with the aim of excluding brain alteration, whereas control subjects without DD and NVIQ above 85 (*n* = 18) performed brain MRI examination because of various reasons. Specifically, the subjects of the latter group performed MRI on the recommendation of the general paediatrician for: headache (*n* = 5), seizures with fever (*n* = 3), strabismus (*n* = 3), cataract (*n* = 2), recurrent episodes of dizziness (*n* = 2), minor head trauma (*n* = 2) and diplopia (*n* = 1). We included within the control group subjects who have undergone MRI for medical reasons since ethical considerations did not allow us to perform a MRI scan under sedation with a general aenesthesia in young subjects for research purposes only. At the same time, it is crucial to select control subjects scanned under sedation in order to have MRI scans that are comparable in terms of head motion with the MRI exams of the ASD patients.

All MRI brain scans were recorded as normal by two paediatric neuroradiologists. The composition of our dataset has been summarized in Table 1, where the mean, the standard deviation and the range of age and NVIQ regarding each group have been reported.

**Table 1:** Dataset composition and sample characteristics

Variable		Subject group, mean ± std [range]											
Age (months)		ASD (*n* = 76)				Controls (*n* = 76)							
		53 ± 17 [25–88]				53 ± 18 [22–89]							
NVIQ		71 ± 22 [30–113]				73 ± 23 [35–112]							
ADOS	LING/COM	5.1 ± 1.5 [2–8]											
	INT/SOC	8.6 ± 2.6 [4–14]											
	TOT	13.7 ± 6.7 [6–21]											
CSS		6.1 ± 1.8 [2–10]											
Age (months)		Males (*n* = 38)		Females (*n* = 38)		Males (*n* = 38)		Females (*n* = 38)					
		53 ± 16 [27–87]		53 ± 18 [25–88]		53 ± 17 [24–88]		53 ± 19 [22–89]					
NVIQ		71 ± 21 [39–113]		71 ± 22 [30–103]		74 ± 23 [43–112]		71 ± 24 [35–100]					
ADOS*	LING/COM	5.0 ± 1.5 [2–8]		5.2 ± 1.4 [2–8]									
	INT/SOC	8.5 ± 2.6 [4–14]		8.8 ± 2.7 [4–14]									
	TOT	13.5 ± 3.6 [7–21]		14.0 ± 3.8 [6–20]									
CSS		6.0 ± 0.9 [3–10]		6.3 ± 0.8 [2–10]									
		DD (*n* = 19)	no-DD (*n* = 19)	DD (*n* = 19)	no-DD (*n* = 19)	DD (*n* = 19)	no-DD (*n* = 19)	DD (*n* = 19)	no-DD (*n* = 19)	ANOVA			
Age (months)		55 ± 16 [27–82]	52 ± 16 [34–87]	47 ± 18 [25–83]	59 ± 16 [36–88]	52 ± 13 [29–72]	54 ± 21 [24–88]	51 ± 18 [26–77]	56 ± 20 [22–89]	ASD-DDm, ASD-DDf, ASD-no-DDm, ASD-no-DDf, C-DDm, C-DDf, C-no-DDm, C-no-DDf			
										**F**		**p**	
										0.8		0.59	
NVIQ		53 ± 9 [39–68]	89 ± 13 [70–113]	53 ± 14 [30–69]	90 ± 10 [71–103]	52 ± 7 [43–75]	95 ± 10 [78–112]	50 ± 9 [35–65]	93 ± 10 [78–100]	ASD-no-DDm, ASD-no-DDf, C-no-DDm, C-no-DDf		ASD-DDm, ASD-DDf, C-DDm, C-DDf	
										*F*	*p*	*F*	*p*
										1.14	0.34	0.33	0.8

*Abbreviations*: *ASD* autism spectrum disorder, *NVIQ* non-verbal intelligence quotient, *ASD-DDm* ASD males with developmental delay, *C-DDm* control males with developmental delay, *ASD-noDDm* ASD males without developmental delay, *C-noDDm* control males without developmental delay, *ASD-DDf* ASD females with developmental delay, *C-DDf* control females with developmental delay, *ASD-noDDf* ASD females without developmental delay, *C-noDDf* control females without developmental delay, *std* standard deviation, *CSS* Calibrated Severity Score

*Information unavailable for five ASD female subjects

This study was approved by the Institutional Review Board of the Clinical Research Institute for Child and Adolescent Neurology and Psychiatry, and the written informed consent was obtained from all parents or tutors.

### Clinical procedures and measures

Patients with ASD were evaluated by a multidisciplinary team, including a senior child psychiatrist, a child psychologist, a speech-language pathologist and an educational therapist, during 5–7 days of extensive assessment. Participants with ASD met diagnostic criteria on the Autism Diagnostic Observation Schedule, and by expert clinical judgement (R.T. and A.C.). In particular, all patients performed the following evaluations.

### Cognitive assessment

A number of well-standardized tests were used to assess intellectual abilities due to differences in the age, verbal skills and functioning level of children. These included: the Leiter International Performance Scale-Revised [53], the Griffiths Mental Development Scale [54], the Italian version of Wechsler Preschool and Primary Scale of Intelligence (WPPSI) [55] and Wechsler Intelligence Scales for Children-Revised (WISC-R) [56]. When the tool provides a mental age (MA), NVIQ was estimated dividing MA by the child’s chronological age (CA): ([MA/CA] × 100). For this study, we consider the NVIQ scores.

### Standardized assessment of autistic symptoms

The Autism Diagnostic Observation Schedule-Generic (ADOS-G) [57] was used to assess social and communicative functioning in children suspected of having an ASD. Module 1 of the ADOS (designed for preverbal children or children with only single words) or Module 2 (children with consistent phrase speech) was administered by two clinical psychologists (F.A. and A.N.) who met standard requirements for research reliability. A calibrated ASD severity score (CSS) was calculated based on ADOS raw scores [58]. The CSS range is 1–10; it provides a measure of autism symptoms that is independent of age and language ability and thus is better suited than the ADOS for assessing the severity of ASD [59]. The average value, the standard deviation and the range of the ADOS and the CSS scores have been reported in Table 1 for the entire sample of subjects affected by ASD and for the male and female subsamples separately.

### MRI data acquisition

All MRI data were acquired in the same tertiary care hospital using a 1.5-T MR Neuro-optimized System (GE HealthCare, USA) fitted with 40-mT/m high-speed gradients. The standard MR protocol for children included a whole brain Fast Spoiled Gradient Recalled acquisition in the steady-state T1-weighted series (FSPGR). Isotropic images were collected in the axial plane with repetition time 12.4 ms, echo time 2.4 ms, inversion time 700 ms, flip angle of 10°, yielding 124 contiguous 1.1-mm slices with an in-plane resolution of 1.1 × 1.1 mm^2^. All children were sedated with a general anaesthesia with a halogenated agent while spontaneously breathing.

### Data analysis procedure

We implemented an algorithm pipeline based on innovative data analysis techniques able to highlight the tiny brain anatomical abnormalities that characterize patients with ASD with respect to controls and then reveal gender-specific effects.

**Fig. 1:**
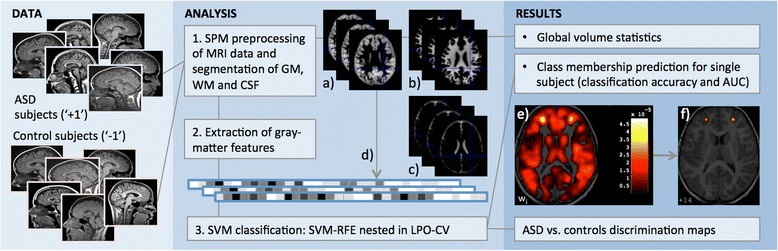
Sketch of the data analysis procedure. the MRI data of patients with autism spectrum disorder (ASD) and matched control subjects are preprocessed with the Statistic Parametric Mapping (SPM) software package to obtain the segmented grey matter (**a**), white matter (**b**), cerebrospinal fluid (**c**); the statistical analysis is performed on global volumes; the GM tissue of each subject is rolled down into a vector of features (**d**) and processed by a machine-learning analysis based on support vector machines (SVMs); to reduce the data dimensionality the recursive feature elimination (RFE) procedure nested in leave-pair-out cross validation (LPO-CV) is implemented during the SVM training. The SVM-based procedure has two main goals: to allow making a prediction of ASD diagnosis on unknown cases (subjects that have not entered the SVM training phase), with classification performance estimated in terms of either the accuracy or the area under the receiver operating characteristic curve (AUC); to directly extract a map encoding the anatomical differences between the patients with ASD and the matched control subjects (discrimination maps). The discrimination maps are shown in the figure at the beginning (**e**) and at the end (**f**) of the SVM-RFE procedure

As sketched in Fig. 1, starting from a sample of sMRI of accurately matched patients with ASD and control subjects, the image preprocessing step is applied to segment the brain data into the different cerebral tissues. Their volumes are investigated for global anatomical abnormalities. Then, the GM tissue is examined at the voxel level through a machine-learning technique based on SVM with two main goals: (1) to evaluate the predictive power of sMRI in ASD; (2) to identify and localize possible GM differences between subjects with ASD and controls. SVMs allow making a prediction of diagnosis on unknown cases (subjects that have not entered the SVM training phase), i.e. the single-subject classification. The performance of the SVM classifier, which is typically provided in terms of the accuracy of the algorithm, provides also an indication of how much “separable” are the two-class of subjects (i.e. ASD/female ASD/male ASD patients from matched controls). It is possible to directly extract a map of the anatomical differences that are responsible for the two-class separation, i.e. the brain areas more involved in the pathology. In addition, the SVM classifier assigns to the test cases a continuous output, whose sign determines the class prediction and whose value can be investigated for correlation with the autistic symptoms of subjects.

The analysis pipeline has been implemented first on the entire data set, and then separately on the male and female subgroups to detect those gender-related alterations that differentiate patients with respect to control subjects.

### Image preprocessing

The sMRI of all subjects of the study were preprocessed using the Statistical Parametric Mapping (SPM) software package SPM8 (Wellcome Department of Imaging Neuroscience, London, UK, http://www.fil.ion.ucl.ac.uk/spm). The Diffeomorphic Anatomical Registration using Exponentiated Lie algebra (DARTEL) algorithm [60] was implemented, involving a diffeomorphic warping to achieve an accurate inter-subject registration and to generate population-based templates, which is particularly recommended when dealing with a population of children. Such a procedure led to the creation of a template specific of the whole data set and of two templates for the male and female subgroups.

The preprocessing [40] included the following steps: (1) checking for scanner artifacts and gross anatomical abnormalities for each subject; (2) setting the image origin to the anterior commissure; (3) segmentation of brain tissues using “New Segment” SPM tool; (4) importing the parameter files produced by tissue segmentation in the DARTEL procedure to generate a population-based template; (5) modulation to take into account volume differences in tissue segments; (6) warping of the segmented brain tissues into the Montreal Neurological Institute (MNI) space [60]; and (7) checking for homogeneity across the sample and using standard smoothing (i.e. with 8-mm isotropic Gaussian kernel). After this preprocessing, smoothed modulated normalized data (in the MNI space) were obtained to be used for the volumetric analysis and for the SVM pattern classification.

### Global volumetric analysis of brain GM segments

Group differences were evaluated for GM, WM, cerebrospinal fluid (CSF) absolute volumes, and total intracranial volume (TIV), obtained in the brain segmentation step of the image preprocessing. The TIV was calculated as the sum of GM, WM and CSF volumes. The analysis of variance (ANOVA) was applied to identify any significant difference between patients with ASD and healthy subjects in global tissue volumes.

### Pattern classification approach: SVM analysis and discrimination maps

We followed the whole-GM classification approach with SVM proposed in [41, 61] and applied in [44, 45], using the SVM-Light software package developed by Thorsten Joachims (http://svmlight.joachims.org), which is freely available for scientific and non-commercial use [62, 63]. The pattern classification techniques are multivariate and thus they take into account specific inter-regional dependencies.

A SVM [37] is a supervised classification method, i.e. it requires a training set, where to learn the differences between the two groups, and a test set to evaluate the classification performance on previous unseen data. In the machine-learning framework, each image is treated as a point in a high-dimensional space, where the space dimension is equal to the number of voxels in the segmented GM image. The sequences of the intensity values of the GM segments obtained in the image preprocessing were given in input to the SVM as vectors of features (more than 5 × 10^5^). The elements of these vectors represent the amount of GM in each voxel, as the modulation step has been implemented in the image preprocessing. Appropriate labels have been appended to the feature vectors categories of patients and controls.

In our analysis, linear-kernel SVMs have been employed for image classification. Training a SVM involves the estimation of the largest-margin hyperplane that separates the training examples, where the margin represents the distance from the separating hyperplane to the closest training examples. It has been demonstrated by Vapnik [37] that a larger margin corresponds to greater generalization performance of the SVM classifier. The training examples that lie on the margin are called support vectors. The separating hyperplane **w ×** + b = 0 is identified by the weight vector **w** and the offset b, where **w** is a linear combination of the support vectors, and it is normal to the hyperplane. We have chosen to implement linear-kernel SVM as the weight vector **w** indicates the direction along which the images of the two groups differ most, and it can be directly represented as a map, which is referred to as the discrimination map.

The SVMs have been trained according to the leave-pair-out cross validation (LPO-CV) technique; hence, excluding one couple of matched subjects from the training set at each iteration, and testing the trained SVM on it, this method provides an unbiased estimate of the performance of a classifier when a rather limited dataset is available. In addition to the classification accuracy (percentage of correctly classified subjects), we evaluated the receiver operating characteristic (ROC) curve [64], where the sensitivity (true positive ratio, i.e. the percentage of patients with ASD correctly classified as ASD) is plotted vs. the false positive ratio (i.e. the percentage of misclassified control subjects). From the ROC curve, we estimated the area under curve (AUC), whose meaning has been proved to be the probability that a random pair of positive/diseased and negative/non-diseased individuals are correctly identified by the diagnostic test [65].

We implemented as a data dimensionality reduction procedure to identify the voxels with the highest discriminating power the SVM recursive feature elimination (SVM-RFE) algorithm [66, 67]. This method iteratively removes features (voxels) from the data set with the purpose of eliminating the major number of non-informative features/voxels (low |w_i_|) while retaining the most discriminating ones (high |w_i_|). The SVM-RFE algorithm was applied within the LPO-CV framework: a new SVM classifier was trained at each step of the LPO-CV by iteratively reducing the number of retained voxels in the data according to the ranking criterion based on |w_i_|. In the current study, the supervised machine-learning approach was not mainly employed with the aim of predicting the class membership of undiagnosed subjects, but as a multivariate technique to estimate the sample separation ability of the discrimination maps containing the between-group structural differences. At each step *j* of the recursive algorithm, we retained the *n*_*j*_ most discriminating voxels/features, with *j* = *1*, *2*, …*N*, where *N* indicates how finely the AUC vs. the number of retained voxels is sampled. Linear-kernel SVMs depend only on one parameter (C), that regulates the trade-off between having zero training errors and allowing for misclassifications. The heuristic estimate of the C parameter ($C = N/{\displaystyle {\sum}_{i=1}^N{\underline{x}}_i\cdot {\underline{x}}_i}$ where ${\underline{x}}_i$ for *i* = *1*, .., *N* are the *N* training vectors) has been adopted in the present study.

Patients and controls labels in our sample are “+1” and “−1”, respectively. Since the weight vector is estimated in such way that it points to the positive training examples, a positive value in the discrimination map means relatively higher GM volume in patients with respect to controls, and a negative value means relatively higher GM volume in controls than in patients [41].

### Correlation between global and local brain volumes, SVM test margins and diagnostic criteria

We investigated the possible correlation of the autistic symptoms with the global brain volumes (GM, WM and CSF), with the local GM changes and with the SVM test margins (i.e. the classification output). The latter consisted in the output obtained for each patient during the test phase in LPO-CV of the SVM-RFE algorithm applied on the entire dataset and on the gender-related subgroups. As a parameter indicating the ASD symptom severity, we considered the ADOS total scores. Five female subjects of the dataset were excluded from this test because of the absence of their ADOS score. The Pearson’s correlation index was estimated to investigate the evidence against the null hypothesis of no correlation.

## Results

### Participant characteristics and volumetric analysis

Age and NVIQ of the entire sample of 76 males and 76 females (males: 38 ASD and 38 controls; females: 38 ASD and 38 controls), of male and female subgroups separately, and finally, the characteristics of two subgroups with and without DD in male and female subsets are reported in Table 1. Subgroup homogeneity with respect to age and NVIQ was evaluated according to the ANOVA test. No significant differences were detected between groups on *p* < 0.05 relative to age when the ANOVA test was applied on all the subgroups composing the dataset, obtaining *F* = 0.8, *p* = 0.59, with ν_1_ = 7 degrees of freedom for the between group variance, ν_2_ = 144 degrees of freedom for the within group variance. The same test was applied on the subgroups of subjects with and without DD, obtaining no significant differences (*F* = 1.14 and *p* = 0.34 with ν_1_ = 3 and ν_2_ = 72 for subjects without DD; *F* = 0.33 and *p* = 0.80 with ν_1_ = 3 and ν_2_ = 72 for DD) in age and NVIQ.

The absolute volumes of GM, WM, CSF and TIV for each subject of the data set, calculated from the segmented brain images generated during the image preprocessing were considered. In Table 2, the means and the standard deviations of GM, WM, CSF volumes and TIV are displayed, and the between-group ANOVA statistics are reported.

**Table 2:** Global brain volumes of ASD subjects and controls in the entire dataset, in male and female subgroups, and in DD and no-DD male (m) and female (f) subsets

Variable (ml)	Subject group, mean ± std								ANOVA							
	ASD (*n* = 76)				C (*n* = 76)				ASD vs. C							
									*F*				*p*			
GM (ml)	662 ± 67				629 ± 80				7.39				0.007			
WM (ml)	424 ± 47				400 ± 55				8.84				0.003			
CSF (ml)	225 ± 25				218 ± 34				2.41				0.123			
TIV	1310 ± 130				1250 ± 160				7.18				0.008			
	ASDm (*n* = 38)		ASDf (*n* = 38)		Cm (*n* = 38)		Cf (n = 38)		ASDm vs. Cm				ASDf vs. Cf			
									*F*		*p*		*F*		*p*	
GM (ml)	699 ± 62		625 ± 48		664 ± 83		594 ± 60		4.25		0.043		5.90		0.018	
WM (ml)	449 ± 43		400 ± 38		422 ± 56		376 ± 44		5.38		0.023		5.93		0.017	
CSF (ml)	238 ± 25		212 ± 17		232 ± 38		203 ± 19		0.52		0.470		5.08		0.027	
TIV (ml)	1390 ± 120		1240 ± 100		1320 ± 170		1170 ± 120		3.90		0.052		6.13		0.016	
	ASD-DDm (*n* = 19)	ASD-noDDm (*n* = 19)	ASD-DDf (*n* = 19)	ASD-noDDf (*n* = 19)	C-DDm (*n* = 19)	C-no-DDm (*n* = 19)	C-DDf (*n* = 19)	C-no-DDf (*n* = 19)	ASD-DDm vs. C-DDm		ASD-noDDm vs. C-noDDm		ASD-DDf vs. C-DDf		ASD-noDDf vs. C-noDDf	
									*F*	*p*	*F*	*p*	*F*	*p*	*F*	*p*
GM (ml)	702 ± 85	696 ± 65	616 ± 59	633 ± 35	661 ± 85	668 ± 83	584 ± 53	605 ± 66	2.98	0.09	1.34	0.255	3.21	0.081	2.73	0.107
WM (ml)	453 ± 44	445 ± 43	393 ± 44	406 ± 31	424 ± 62	421 ± 51	369 ± 41	384 ± 47	2.72	0.108	2.55	0.119	3.04	0.090	2.90	0.097
CSF (ml)	238 ± 31	238 ± 28	211 ± 20	213 ± 13	228 ± 31	236 ± 44	200 ± 16	206 ± 22	1.13	0.294	0.01	0.906	3.54	0.068	1.65	0.207
TIV (ml)	1390 ± 210	1380 ± 130	1220 ± 120	1250 ± 70	1310 ± 170	1330 ± 170	1150 ± 110	1200 ± 130	2.71	0.109	1.24	0.272	3.37	0.075	2.78	0.104

*Abbreviations*: *ASD* autism spectrum disorder, *C* controls, *ASDm* ASD males, *Cm* control males, *ASDf* ASD females, Cf control females, *ASD-DDm* ASD males with developmental delay, *C-DDm* control males with developmental delay, *ASD-noDDm* ASD males without developmental delay, *C-noDDm* control males without developmental delay, *ASD-DDf* ASD females with developmental delay, *C-DDf* control females with developmental delay, *ASD-noDDf* ASD females without developmental delay, *C-noDDf* control females without developmental delay, *GM* grey matter, *WM* white matter, *CSF* cerebrospinal fluid, *TIV* total intracranial volume, *std* standard deviation

We found the following significant differences (*p* < 0.05): GM and WM volumes were found higher in patients with ASD than controls in the entire data set and in the subgroups of male and female subjects; no significant differences between patients with ASD and controls in the subgroups of subjects with and without DD have been found. In addition, these statistical analyses pointed out meaningful differences in TIV in the entire dataset, and in CSF volume and TIV in the female subgroup.

### SVM analysis of GM segments

The GM segments of the subject groups were analysed with the SVM-RFE procedure both for the entire dataset and for the gender-specific subgroups to reduce the total number of GM voxels (more than 5 × 10^5^) to the most informative 1000 voxels. In particular, the SVM-RFE algorithm is nested within the LPO-CV scheme and runs according to the following steps: (1) in the first iteration, the discrimination map with all GM voxels is computed training an SVM on all subject pairs except for the *k*^th^ pair, then the SVM is tested on the left-out pair according to the LPO-CV protocol; (2) for each iteration *j* of the RFE procedure, a new SVM is trained on the *n*_*j*_ features with highest |*w*_*i*_| (with *i* = 1, 2, … *n*_*j*_) values, and it is tested on the *k*^th^ left-out pair, and a discrimination map with reduced number of voxels *n*_*j*_ is generated; (3) the discrimination map obtained at the step *j* is used in the *j* + 1 iteration. At each step of the RFE procedure, the SVM outputs obtained in LPO-CV are used to compute the classification accuracy and the AUC figure of merit, until only the last 1000 most informative voxels are retained. The trend of AUC as a function of the *n*_*j*_ retained during the feature selection RFE procedure are shown in Additional file 2. The significance levels of the figures of merits used to quantify the SVM classification performance have been estimated by the non-parametric permutation test. This method allows to empirically estimate the statistic distribution of the figures of merit under a null hypothesis of no discrimination ability between the two-class labels (corresponding to the chance level expectation value of 0.5 for both the accuracy and the AUC). By permuting the class labels 1000 times randomly and training the SVM with these sets of permutated labels, we estimated a probability distribution of the accuracy and the AUC and calculated the *p* value as the number times the obtained their values outperform the values obtained by using the non-permutated data, divided by the chosen number of permutations. The classification performance thus achieved are AUC_M_ = 0.68 (accuracy = 0.62) on *p* < 0.001 for the male subset, AUC_F_ = 0.57 (accuracy = 0.54) on *p* < 0.05 for the female subset and AUC_MF_ = 0.65 (accuracy = 0.59) on *p* < 0.001 for the entire dataset. It can be noticed that the AUC values obtained are quite modest, underlying a substantial overfitting problem. This is confirmed by the evidence that the SVM classifiers achieve in training definitively better results, i.e., AUC = 0.82, 0.80, 0.77 for the male, for the female subsets and for the whole sample, respectively. Moreover, despite the larger number of training subjects contained in the male and female group with respect to the gender-specific subgroups having the potentiality to lead to a more efficacious SVM training phase and better generalization ability, the best discriminating performance is obtained for the male-only subgroup. The ranking of the three sets of subjects according to the classification ability suggests that males with ASD are more easily distinguishable from controls by the SVM classifier with respect to the fully matched group of female subjects, while the separation ability achieved on the whole dataset coherently positions in between, reflecting the prominent heterogeneity factor introduced by the gender.

### Discrimination maps

**Fig. 2:**
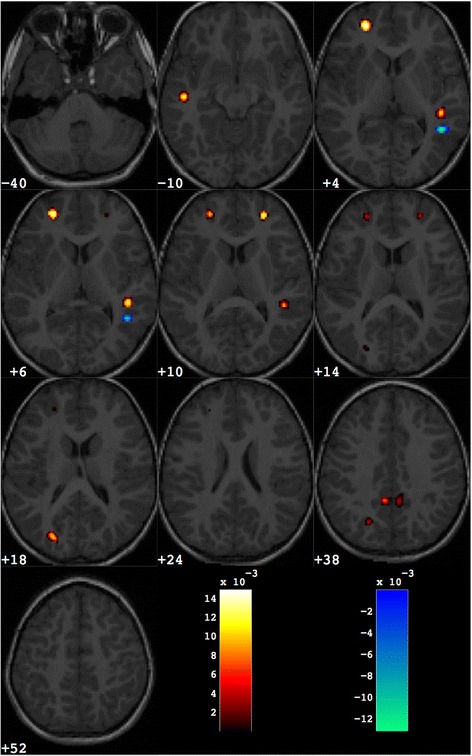
Discrimination map obtained for the entire group of male and female subjects. About 1000 voxels are retained out of the total amount of grey matter voxels (more than 500,000). The map is overlaid to a representative structural MR image. The regions in *red scale* represent the brain areas where grey matter is greater in group with ASD with respect to controls; the regions in *blue scale* are referred to the opposite contrast

The discrimination maps obtained with the SVM-RFE procedure stopped at 1000 retained voxels are as many as the number of pairs contained in each dataset, as they are generated in LPC-CV (i.e. 38 maps for male and female datasets, 76 maps for the entire dataset). The sets of significant 1000 voxels in each map are in principle non consistent across all maps. However, as we observed, a strong similarity among all 1000-voxel discrimination maps, we adopted the restrictive criterion of considering only those regions with a non-null overlap across all maps. We reported in Additional file 2 an example of discrimination maps obtained at different values of *n*_*j*_ obtained during the SVM-RFE procedure. The 1000-voxel discrimination map obtained with the above-described operations for the entire dataset of male and female subjects is reported in Fig. 2. Since it is possible to distinguish between the brain regions where GM is greater or lower in patients with respect to the control group when w_i_ is positive or negative, in Fig. 2 these regions are separated using different colour scales (red and blue, respectively). In Table 3, the Talairach coordinates and the w_i_ value of the centroid of these relevant regions are reported in addition to the anatomic labels assigned.

**Table 3:** Grey matter regions that discriminate between the groups of ASD and control subjects (entire dataset). These are grouped in brain areas where grey matter is greater in ASD with respect to controls (ASD > Controls) and those where grey matter is lower in ASD than controls (ASD < Controls)

Number of voxels	Weight in the centroid	Talairach coordinates			Brain area			
		*x*	*y*	*z*				
ASD > controls								
114	0.0123	−49	−19	−6	L	Temporal Lobe	Superior Temporal Gyrus	BA 20
267	0.0144	−25	47	13	L	Frontal Lobe	Superior Frontal Gyrus	BA 10
128	0.0118	44	−37	7	R	Temporal Lobe	Superior Temporal Gyrus	BA 22
74	0.0120	25	44	18	R	Frontal Lobe	Superior Frontal Gyrus	BA 10
57	0.0104	−26	−80	12	L	Occipital Lobe	Middle Occipital Gyrus	BA 19
128	0.0119	−25	−68	26	L	Parietal Lobe	Precuneus	BA 7
152	0.0111	5	−50	29	R	Parietal Lobe	Precuneus	BA 31
182	0.0107	−7	−47	30	L	Parietal Lobe	Precuneus	BA 31
ASD < Controls								
83	−0.0128	45	−50	4	R	Temporal Lobe	Inferior Temporal Gyrus	BA 37

**Fig. 3:**
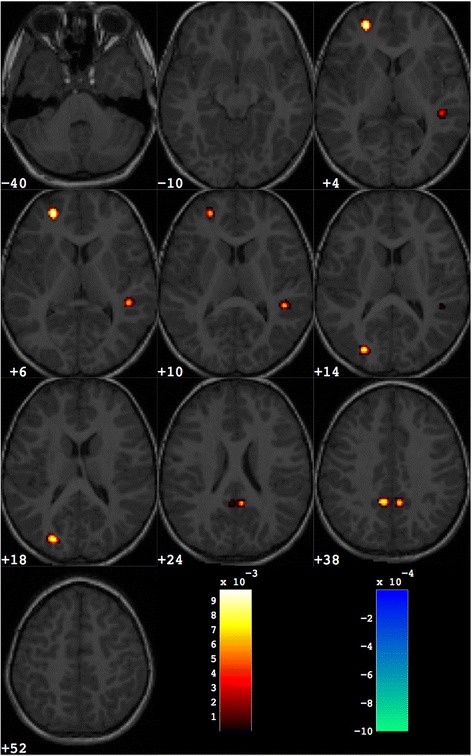
Discrimination map highlighting the differences between male subjects with ASD from matched controls, obtained retaining about 1000 out of the total amount of grey matter voxels. *Colour scale* holds the same meaning as for Fig. 2

**Fig. 4:**
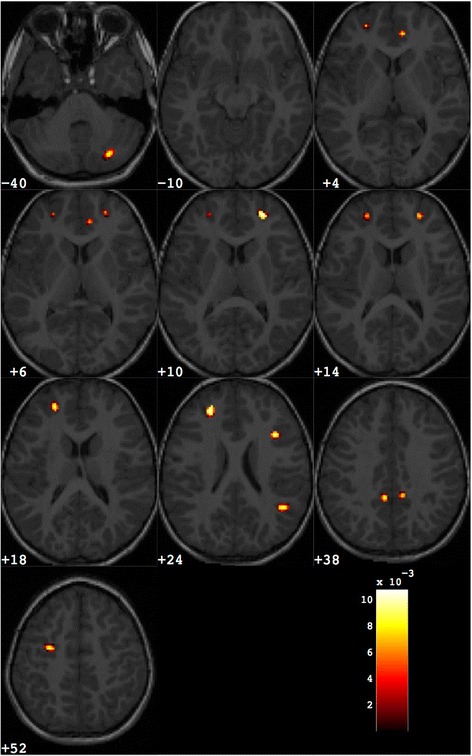
Discrimination map highlighting the differences between female subjects with ASD from matched controls, obtained retaining about 1000 out of the total amount of grey matter voxels. *Colour scale* holds the same meaning as for Fig. 2

The discrimination map obtained for the male group of subjects is reported in Fig. 3. The relevant brain areas are listed in this case in Table 4. For the female group of subjects the discrimination maps and the list of relevant brain areas are reported in Fig. 4 and Table 5, respectively.

**Table 4:** Grey matter regions that discriminate between the groups of ASD and control subjects for male subjects

Number of voxels	Weight in the centroid	Talairach coordinates			Brain area			
		*x*	*y*	*z*				
ASD > controls								
208	0.0097	−25	47	13	L	Frontal Lobe	Superior Frontal Gyrus	BA 10
90	0.0069	44	−38	8	R	Temporal Lobe	Superior Temporal Gyrus	BA 22
132	0.0084	−26	−82	11	L	Occipital Lobe	Middle Occipital Gyrus	BA 19
289	0.0085	5	−50	29	R	Parietal Lobe	Precuneus	BA 31
294	0.0079	−8	−49	30	L	Parietal Lobe	Precuneus	BA 31

**Table 5:** Grey matter regions that discriminate between the groups of ASD and control subjects for female subjects

Number of voxels	Weight in the centroid	Talairach coordinates			Brain area			
		*x*	*y*	*z*				
ASD > controls								
106	0.0088	26	−70	−37	R	Cerebellum, Posterior Lobe	Inferior Semi-Lunar Lobule	
74	0.0077	8	39	11	R	Limbic Lobe	Anterior Cingulate	BA 32
258	0.0090	−25	40	23	L	Frontal Lobe	Middle Frontal Gyrus	BA 9
107	0.0107	24	45	18	R	Frontal Lobe	Superior Frontal Gyrus	BA 10
126	0.0095	43	−54	23	R	Temporal Lobe	Superior Temporal Gyrus	BA 39
87	0.0098	35	14	29	R	Frontal Lobe	Middle Frontal Gyrus	BA 9
107	0.0090	−8	−46	32	L	Parietal Lobe	Precuneus	BA 31
37	0.0087	8	−44	35	R	Limbic Lobe	Cingulate Gyrus	BA 31
99	0.0095	29	−11	48	L	Frontal Lobe	Middle Frontal Gyrus	BA 6

The regions with increased GM obtained in this analysis for the female group are in agreement with the result reported in a previous study on the same dataset [44], where only the most relevant regions corresponding to less than 200 voxels were highlighted and discussed.

**Fig. 5:**
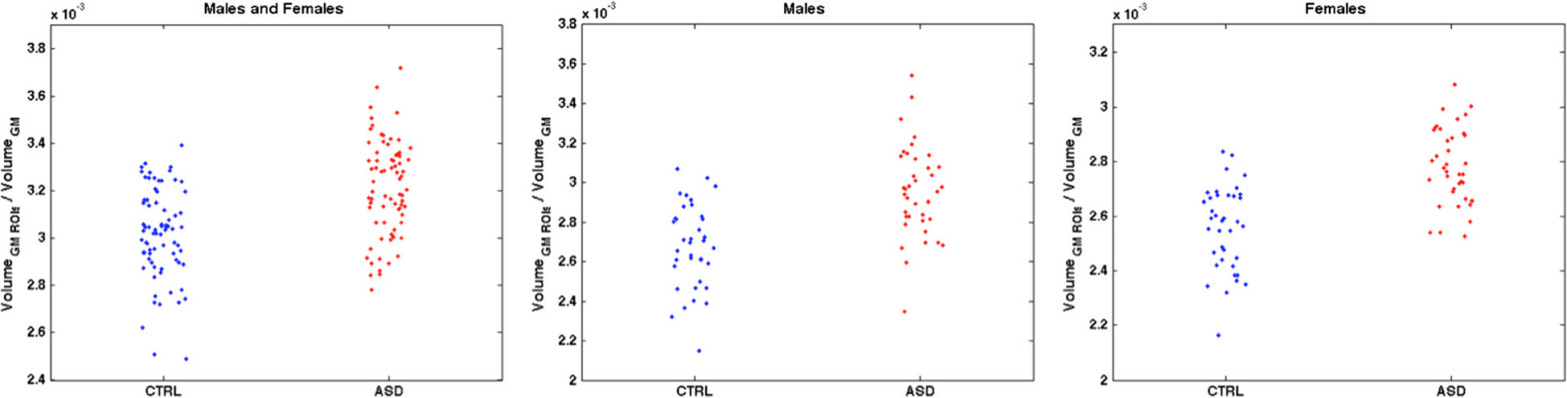
The amount of the GM volumes summed over the small areas of the discrimination maps are shown for patients with ASD and controls for the entire dataset and for the gender-related subgroups. The values are normalized to the global GM volume of each subject. A little noise is introduced on the *x*-axis of the plot to improve the visualization of the distribution

The extent of local GM changes that is responsible for the separation between the patients with ASD and the control subjects is visible in the scatter plots shown in Fig. 5. The amount of the GM volumes summed over the small areas of the discrimination maps are normalized to the global GM volume of each subject and shown for patients with ASD and controls for the entire dataset and for the gender-related subgroups. It can be noticed that the values are widespread within the samples and are not driven by a subset of children.

### Relationship between global and local brain volumes, SVM classification output and level of symptom severity

The possible correlations between the quantitative information derived from sMRI (global GM, WM and CSF volumes, volumes of the GM regions identified by SVM, SVM classification output) and calibrated severity scores [58] derived from the ADOS-G [57] raw total scores were investigated. No significant results were achieved for any of the above-mentioned tests against the null hypothesis of no correlation on *p* < 0.05 for the entire dataset analysis and for the gender-related subgroups.

## Discussion

This study examined the neuroanatomical differences between young children with ASD and carefully matched controls through the SVM multivariate approach applied to sMRI data. The strict patient selection obtained in order to match male and female children with ASD for chronological age, symptom severity and NVIQ allowed us to perform a rigorous analysis of whole brain sMRI data on gender differences in young patients with ASD. To date, two seminal papers [68, 69] examined the structural neuroanatomy of gender differences in adults with ASD. However, different age ranges of the samples as well as methodological differences in the study design and data analysis make a direct comparison with current results difficult.

The current study revealed three major findings: (1) the GM of patients with ASD (males and females) is greater than that of the matched control group in Left (L) and Right (R) Superior Frontal Gyrus (Brodmann –BA– 10); L and R Precuneus (BA 31); L Superior Temporal Gyrus (BA 20); R Superior Temporal Gyrus (BA 22); (2) two clusters of increased GM located in the L middle occipital gyrus (BA 19) and R Superior Temporal Gyrus (BA 22) are specific of males with ASD; and (3) ASD females had larger volume than their counterparts in several clusters involving predominantly the bilateral frontal lobe.

Consistently with these findings, postmortem studies on the neuropathology of ASD indicated focal patches of abnormal laminar cytoarchitecture and cortical disorganization of neurons in prefrontal, temporal and occipital regions in a strong majority of children with ASD [70], and pointed to an altered microanatomical organization in ASD [71].

### Global volume group differences in the entire data set and in male/female subgroups

The results of the ANOVA analysis for group differences revealed that the GM volume, the WM volume and the TIV were significantly higher in patients with ASD as compared to controls.

The increased TIV found in the current study is in agreement with the “extreme male brain –EMB– theory of autism” [72] according to which the brain of patients with ASD represents an atypical extreme of typical male brain [73]. A possible contribution to the neuroanatomical signature of EMB comes from the early altered endocrine profile of patients with ASD; indeed, recent evidence indicates that males subsequently diagnosed with ASD showed elevated levels in utero of sex steroids (progesterone, 17a-hydroxy-progesterone, androstenedione and testosterone), as well as cortisol [74], and that foetal testosterone influences grey matter sexual dimorphism in typical subjects [75]. Future studies that combine the evaluation of prenatal androgen levels with the MRI volumetric data of sexually dimorphic structures in patients with ASD should verify whether a correlation exists between these two variables.

Our findings are in accordance with several sMRI studies showing an enlarged brain volume in ASD toddlers in comparison to matched controls [52, 76, 77] that may underlie a pathological cellular process leading to disruption of cerebral architecture. Specifically, excess neuron numbers or insufficient synaptic pruning have been proposed. This process is thought to lead to an excess of short-range connections and a deficit in long-range connections between brain regions required for higher-order social, emotional and communication functions [33, 78] that in turn alter typical early experience-expectant learning. It is worth noting that the increased early total brain volume, despite being one of the most consistently reported findings in ASD [79], is not constantly observed [24, 80]; the neuroanatomical heterogeneity of autistic condition, the wide range of clinical variables in the samples, as well as the different data analysis techniques could contribute to explain the contrasting results. The increased WM volume we found in young children with ASD mirror similar results obtained in this age window [34, 52, 76, 77, 81, 82] and point to a reduced developmental refinement during an interval characterized by important modification of WM structure in typical development.

By contrast, here we did not identify statistically significant differences in CSF in the ASD group compared to the TD sample. This finding is consistent with a recent study on young siblings of individual ASD in which patients who develop ASD and controls did not differ on the total CSF [83]; conversely, an increased extra-axial accumulation of CSF was found to be a predictive marker of ASD by 6 months of age in subjects at high risk for developing ASD [84].

The examination of gender effects on brain volumes showed that female participants with ASD exhibited significantly more GM (*p* = 0.018), WM (*p* = 0.017), CSF (*p* = 0.027) and TIV (*p* = 0.016) than female controls, whereas the cerebral volumes significantly increased in males with ASD vs. male controls are limited to GM (*p* = 0.043), WM (*p* = 0.023), with a trend towards statistical significance for TIV (*p* = 0.052). Previous evidences on TIV enlargement in young females with ASD are limited and mixed, with an increased total cerebral volume in ASD females [25, 28], or no differences in TIV from controls [24]. In the current study, we have actively excluded all subjects with anthropometric parameters (weight, height and head circumference) lying outside two SD from the mean of normal subjects (see Additional file 1) in order to reduce the etiological heterogeneity of patients with ASD, and to try to enrol patients with idiopathic ASD only. Consequently, individuals with micro (and macro)-cephaly are not included in our final sample. This selection criterion is likely to account for the increased cerebral volumes more evident in the ASD females vs. males with ASD of our sample, since microcephaly is a feature significantly more prevalent in ASD females than in males with ASD in non-selected autistic populations [85].

### Grey matter local volume differences between children with ASD and controls

One of the most remarkable findings in this study concerned the greater GM volume in patients with ASD with respect to control group in the following main significant regions: bilateral precuneus/posterior cingulate cortex, bilateral superior frontal gyrus, bilateral superior temporal gyrus. These structures all belong to the Default Mode Network (DMN) [86], a set of neural regions highly active in the absence of a task, which also include medial prefrontal cortex, parahippocampal gyrus, and lateral parietal cortex/angular gyrus. The dynamic suppression of the DMN is thought to be associated with higher cognitive and emotional processing [87]. A disruption in the DMN has been consistently reported in patients with ASD [88]. However, resting-state fMRI (rs-fMRI) studies investigating the DMN in young children with ASD are limited to a report of reduced functional connectivity between specific ROIs implicated in language processing in toddlers with ASD (age range, 1–3.8 years) [89]. The relevance of DMN regions in the ASD pathogenesis has been reported also in two recent investigations of older subjects [90, 91] in which the best classification accuracy in discriminating adult and adolescent subjects with ASD from control participants was obtained when regions within the DMN were considered. DMN studies in typical individuals described greater GM volume within two nodes of the DMN (posterior cingulate cortex –PCC- and medial prefrontal cortex) in children compared to young adults, suggesting that synaptic pruning during development contributes to DMN maturation [92]. Our findings of an excess of GM in PCC of ASD young children with respect to controls comparable in age invites speculation on a protracted anatomical immaturity of this DMN region in patients with ASD.

Besides their involvement in the DMN, the above-mentioned regions of increased GM in patients with ASD belong to the neural circuitry supporting theory of mind (ToM) [93], the ability to take the perspective of another individual, whose early disruption may contribute to the social and communication impairments in individuals with ASD [94].

The opposite pattern of results (grey matter greater in controls than in individuals with ASD) was observed only in the R inferior temporal gyrus (ITG, BA 37), an area involved in emotional processing and social cognition [95], as well as in multimodal sensory integration [96] and visual perception [97]. An altered visual information elaboration involving ITG activation instead of fusiform activation may be related to a different brain modality of face processing in patients with ASD, as suggested by the seminal work of Schultz et al. [98], in adults with ASD. It is possible that an altered neuroanatomical network comprising ITG would contribute to the reduced attention to faces, a very early and specific symptom of ASD emergence [99]. Ad-hoc brain-behaviour investigations should be performed in order to verify this issue.

### Consistent regions of altered neuroanatomy in male and female children with ASD

Increased GM in the bilateral ventral precuneus/posterior cingulate cortex (PCu/PCC) occurred on both male and female children with ASD with respect to their controls and thus would constitute a gender-independent neural signature of ASD. The PCu/PCC structural alteration possibly relates to atypical socio-cognitive functions in the ASD condition, since this structure is involved in a number of higher-order cognitive processes, including empathy, self/other distinction, episodic memory, and self-representation [100].

Moreover, as reported by a recent meta-analysis on gender differences in human brain morphology, precuneus exhibits sexual dimorphism in TD subjects [101]. In fact, neurotypical male adults showed an increased bilateral PCu/PCC volume in comparison with females. Thus, the enlargement of regional GM volume in the bilateral PCu/PCC of patients with ASD supports the EMB theory.

We also observed larger left middle/superior frontal gyrus (MFG/SFG) (BA9/10) GM volume in males and females with ASD relative to gender-matched controls. The possible impact of MFG/SFG alteration in ASD pathogenesis has been extensively discussed in our previous study [44].

### Gender-specific grey matter local volume differences

#### Distinct and specific brain regions in males with ASD

Two areas of increased GM volume in the left middle occipital gyrus (BA 19) and R superior temporal gyrus (STG; BA 22) are typical to the subgroup of males with ASD. The involvement of the occipital region was quite unexpected, since, according to an anteroposterior gradient of abnormal brain growth trajectories in ASD, the frontal and the temporal lobes are more implicated in the early cerebral overgrowth, whereas the occipital regions are least affected [27, 52, 81]. Also at the microscopic level, histologic studies did not provide evidence for significant alterations in this area in children with ASD [102]. Analogously, some of the neuropsychological functions sustained by the occipital lobe (e. g. visual detail perception) are frequently preserved or even enhanced in ASD individuals, especially in the case of high-functioning subjects (for review, see [103]). Of note, a clinical report on gender differences found that visual reception skills were more impaired in male than in female toddlers with ASD (age range, 18–33 months) [17]. Nevertheless, how the atypical volume of BA 19 we detected in males with ASD relates to behavioural abnormalities requires a further and tailored investigation that combines brain MRI and clinical measures.

We also observed increased volume in the R STG, a cortical area implicated in high-level integration of perceptual multimodal information [104]. Abnormalities in the STG are considered relevant to the ASD pathogenesis because of its important role in processing language and social stimuli [105]. Specifically, the R STG, besides its specific role in social perception, is involved in the processing of semantic [106] and prosodic information [107], two aspects of language often impaired in patients with ASD [108]. Our findings of increased GM volume in R STG were consistent with a previous investigation focused on R STG of children and adolescents with ASD (age range, 8.8–18.3 years) [109], and extend into a younger age the stage at which this volumetric difference can be detected. In a similar vein, a recent VBM study in toddlers with ASD (mean age, 30 months) relative to matched subjects with developmental delay localized a GM increase in the R STG [77]. Interestingly, an fMRI investigation [110] and a rs-fMRI study [89] supported STG involvement at very early stages of ASD development (age range, 1–4 years) suggesting its central role in altered neural circuitry of patients with ASD. In fact, the STG is also part of the neural systems underlying the social information processing (social perception, action observation, and theory of mind), and it is a crucial region for the Responding to Joint Attention ability, i.e. the child’s capacity to follow the direction of gaze, head turn and/or pointing gesture of another person, whose failure is indicative of early social-communication impairment and possibly of ASD [111, 112].

#### Distinct and specific brain regions in females with ASD

Females with ASD differed from their controls in regions located primarily in the frontal lobes (bilateral middle frontal gyrus, BA 9; right superior frontal gyrus, BA 10; anterior cingulate, BA 32) with both hemispheres involved. Neuropathological studies confirmed microanatomical alterations in these regions, such as significantly smaller neurons in superior and middle frontal gyrus [113], and significant pyramidal neurons volume deficit in anterior midcingulate cortex of patients with ASD [114]. Moreover, ASD females showed widespread areas with a significant likelihood of GM increases in comparison to males with ASD. Thus, a neuroanatomical dimorphism in young ASD patients was detected. Specifically, males with ASD tended to have broader regions of brain abnormalities relative to typical males, whereas ASD females are characterized by multiple and localized areas of cerebral involvement with respect to female counterparts. Consequently, a different, but not necessarily more impaired brain profile characterized females compared to males with ASD. These findings did not support the “female protective effect”, according to which females have a lower biological vulnerability to developing an ASD and thus potentially require higher brain alterations to reach the threshold of clinical manifestations of an ASD [115].

The clusters of increased GM include those reported in a previous analysis on the same sample of ASD female patients [44]. In fact, the SVM classification procedure adopted in the two studies are similar, but in this study, the GM segments have been obtained by using the *new segment* algorithm of the SPM packages. In addition, the differences that are evident while comparing the discrimination maps presented in the two studies are actually due to the fact that the SVM-RFE maps are shown at two different operative points, allowing the visualization of additional small regions in the present study.

Significantly increased GM volumes in the right anterior cingulate cortex (ACC; BA 32) and right cerebellum were typical of ASD females only. The structural abnormalities in the ACC peculiar to ASD females suggest a sexually dimorphic involvement of this part of the limbic lobe in patients with ASD. Specifically, the ACC is implicated in affective processing [116], as well as in attention elaboration and regulation [117], whose disruption contributes to the social-cognitive and emotional impairment typical of patients with ASD [118].

Of note, some clinical studies on gender differences in comorbid features of patients with ASD evidenced that females exhibited greater internalizing problems, such as sleep difficulty, anxious or depressed affect [18, 119] than males. In this perspective, our findings of ACC volumetric increase specific of females with ASD propose a link between neuroanatomical and phenotypic gender differences that should be confirmed with ad-hoc investigations.

The increased cerebellum volume found only in female patients could point to distinct neuroanatomical substrates underlying ASD in the two genders. A previous meta-analysis of brain structural abnormalities in ASD found an opposite result [120], since as the number of ASD females included in the samples increased, greater reduction in cerebellar volume was detected. However, the small sample size of ASD females, the frequent involvement of older subjects, and the absence of IQ-matched controls of those studies hamper a direct comparison with the current investigation. Despite a recent consensus paper highlighting the neuroanatomical alterations of the cerebellum in autism [121], previous research has produced conflicting results. In fact, increased [25], decreased [122] and preserved volumes [52] have been shown in studies on cerebellar structures in preschoolers with ASD compared to controls. We found an enlarged GM volume located in the posterior lobe of the cerebellum, a region implicated in cognitive and language tasks [123]. Thorough cognitive and linguistic assessments of patients with ASD will now be essential to delineate the specific brain-behaviour relationships of cerebellar abnormalities.

### Limited predictive power of sMRI data

As a complementary result to the discrimination maps derived in the SVM-based analysis, we reported the classifier performance in terms of accuracy and AUC values. It is undoubted that such modest discrimination performance (slightly above the chance level) can be of limited diagnostic utility. This result reflects the subtlety of the morphometric brain alterations in ASD, as well as its heterogeneity. Despite many studies reported higher accuracy values, as reviewed in [51], when the SVM classifiers are trained and tested according to rigorous cross validation procedures and on data samples representative of the ASD population, the accuracy values lie extremely low. A recent result in this direction is the modest accuracy values (<60 %) reported by Haar et al. [124] on a very large data sample of patients with ASD extracted from the ABIDE initiative [125]. Whereas Haar et al. considered the population of males with ASD between 6 and 35 years in comparison to matched controls, our study extends their results to young children in the 2–7 year range, equally distributed between male and female subjects. Consistently, even in a narrower age range, we found that the ASD heterogeneity nearly overwrites the subtle anatomical differences between patients with ASD and matched controls, leading to slightly above chance sample separability with multivariate analysis techniques.

### Can SVM classification provide additional information about sample separability in cross-comparing population cohorts?

As an additional result derived from the SVM-RFE procedure, we notice that the patients with ASD can be distinguished from the matched control population with higher values of both accuracy and AUC for the male cohort with respect to the female cohort, while the performance in the classification of the entire dataset positions in between. It means that the neuroimaging signature distinguishing the ASD population with respect to matched controls are far more evident in the male cohort with respect to the female cohort, considering the same potential confounding variables (i.e. age, NVIQ). As suggested by a recent paper [85], it is possible that the neurological phenotypes of the autistic disorder in females are more heterogeneous than in males, since they derive from different and multiple etiologies. According to this view, the increased etiopathogenic heterogeneity could mirror heterogeneity at the neuroanatomical level that, in its turn, contributes to a more difficult distinction of ASD females from their control group with respect to males with ASD vs. matched controls.

### Correlations between SVM test margin and the calibrated severity score

In the present study, we did not find a significant correlation between the severity of ASD symptoms (assessed with the calibrated severity score [58] derived from the ADOS raw scores) and the classification output assigned by the SVM to each patient with ASD. This result is actually not unexpected as we implemented the LPO-CV procedure to estimate the classifier performance. In this case, all but two subjects are used at each turn to train the SVM and find the optimal hyperplane, which is used to predict the output on the left-out examples. While this procedure is recognized to provide an unbiased estimate of the generalization performance of the SVM classifier, the SVM outputs assigned to the patients with ASD are not computed in relation to the same decision boundary, thus are not comparable. Different strategies could be implemented to study the correlation between indices of symptom severities and candidate neuroimaging biomarkers for a specific pathology, e.g. computing abnormality indices with respect to a reference population through SVM approaches based on unsupervised learning [126, 127].

### Limitations and future directions

There are several limitations to this study. We targeted our SVM analysis on volumetric differences of the GM. Localization of WM differences between patients and controls is beyond the scope of this study and deserves careful investigation in future research with more sensitive and appropriate techniques (e.g. DTI). We restricted our investigation to the comparison of male and female patients with ASD with their gender-matched controls, matched also for chronological age and NVIQ. Another interesting issue of inquiry involves the identification of typical sex differences (i.e. female vs. male controls), with the aim to evaluate their possible overlap with atypical brain regions in females and/or males with ASD, as performed by Lai et al. on adults [69]. Future investigations with an adequately populated sample of TD controls should be planned with the aim to test this methodology also in children with ASD.

A possible limitation of this study could be the inclusion of subjects with DD within the control group, which leads to an increase of its heterogeneity. However, this choice is motivated by the aim of obtaining a larger dataset and a close match for NVIQ between patients and controls, which is known to be a crucial parameter for a reliable interpretation of imaging data [128]. In case-control studies such as ours, excluding DD subjects from the control sample means focusing only on high-functioning patients with ASD. In this case, a large part of the spectrum would be neglected.

We examined young children with ASD presenting to an ASD unit in a large tertiary hospital and research university that receives patients from all over Italy. Consequently, results from this study may not generalize to children with ASD who are not referred for ASD diagnosis until later ages or children diagnosed outside of a specialty ASD unit. In addition, an in-depth examination of other phenotypic features including adaptive functioning, language level, restricted/repetitive behaviours, psychiatric/internistic comorbidities should be performed in order to analyse further possible brain-behaviour correlations. Also, additional information regarding familial status, including parental level of education, parental NVIQ and SES could be useful to investigate their potential influence on children’s brain structure. In addition, the inclusion of other information in the classifiers (e.g. DTI, proton spectroscopy and rs-fMRI, as well as genotypes) would improve the global accuracy of results.

Finally, in order to investigate the degree of specificity of ASD brain distinctive pattern, future studies should include subjects with other neurodevelopmental disorders that are considered in the ASD differential diagnosis (e.g. attention deficit hyperactivity disorder, language impairments, regulatory disorders).

## Conclusions

A considerable difference in regional brain structure that distinguished ASD, males and females, from gender-, age- and NVIQ-matched controls is reported. These differences substantiate the view that a neuroanatomical sexual dimorphism is present in children with ASD. These data suggest that it is important to set up an experimental design that takes gender into account when studying the structural neuroanatomy of ASD, since neuroimaging findings from investigations mostly or only with males (i.e. the majority of research studies so far reported) may not necessarily extend to females. Finally, our research could pave the way for future studies on the gender-related brain-behaviour relationships, with the final aim to contribute to gender-specific diagnostic criteria, symptom profile evaluation and treatment strategies.

## Additional files

Additional file 1:
**Document S1.** Inclusion and exclusion criteria for the participants included in the present study. (DOCX 74 kb) Additional file 2:
**Document S2.** Implementation details of the SVM-RFE procedure. (DOCX 286 kb)

## Abbreviations

ADOS-G: autism diagnostic observation schedule-generic
ANOVA: analysis of variance
ASD: autism spectrum disorder
AUC: area under curve
BA: Brodmann area
CSF: cerebrospinal fluid
CSS: calibrated severity score
DARTEL: diffeomorphic anatomical registration using exponentiated Lie algebra
DD: developmental delay
DMN: default mode network
DTI: diffusion tensor imaging
fMRI: functional MRI
GM: grey matter
IQ: intelligence quotient
ITG: inferior temporal gyrus
L: left
LPO-CV: leave-pair-out cross validation
MFG: middle frontal gyrus
MNI: Montreal Neurological Institute
MRI: magnetic resonance imaging
NVIQ: non-verbal IQ
PCC: posterior cingulate cortex
PCu: Precuneus
R: right
RFE: recursive feature elimination
ROC: receiver operating characteristic
rs-fMRI: resting-state fMRI
sMRI: structural magnetic resonance imaging
SFG: superior frontal gyrus
STG: superior temporal gyrus
SPM: statistical parametric mapping
SVM: support vector machine
TIV: total intracranial volume
ToM: theory of mind
VBM: voxel-based morphometry
WM: white matter
