# Supplementary material for: The effect of gender on the neuroanatomy of children with autism spectrum disorders: a support vector machine case-control study
Source: Mol Autism. 2016 Jan 19;7:5. doi: 10.1186/s13229-015-0067-3 (PMC4717545; doi:10.1186/s13229-015-0067-3)
Supplement: Additional file 1: — Document S1. Inclusion and exclusion criteria for the participants included in the present study. (DOCX 74 kb) [file 13229_2015_67_MOESM1_ESM.docx]

**Exclusion criteria adopted to populate the ASD and the control data samples, rephrased from Calderoni *et al.* (Neuroimage 59, 1013-1022, 2012).**

Exclusion criteria for ASD subjects included: 1) anomalies detected by MRI; 2) for children under 48 months, lack of follow-up after 48 months of chronological age confirming the clinical diagnosis of ASD; 3) neurological syndromes or focal neurological signs; 4) dysmorphic features suggestive of a genetic syndrome; 5) significant sensory impairment (e.g., blindness, deafness); 6) anthropometric parameters (weight, height and head circumference) lying outside two SD from the mean of normal subjects; 7) anamnesis of birth asphyxia, premature birth, head injury or epilepsy; 8) use of any psychotropic medication; 9) presence or history of any other axis I mental disorder and 10) insufficient image quality. The control group was selected so as to meet the same exclusionary criteria as the ASD (except the second criterion specified above) with the further requirements of no family history of ASD (assessed in both DD and noDD) and a score below 20 on the CARS, available for DD patients only. The diagnosis of idiopathic DD was performed after a 5–7 days of thorough multidisciplinary assessment when the mental retardation (IQ<70) remains of unknown etiology after an exhaustive investigation (both clinical and laboratoristic) for underlying causes. In fact, ASD and DD patients performed also the recommended laboratory tests to rule out medical causes of ASD/DD, including audiometry, thyroid hormone disorders, high-resolution karyotyping, DNA analysis of FRA-X and screening tests for inborn errors of metabolism (plasma and urine aminoacid analysis, urine organic acid measurement, urine mucopolysaccarides quantitation, plasma and urine creatine and guanidinoacetate analysis).
