# Supplementary material for: The effect of gender on the neuroanatomy of children with autism spectrum disorders: a support vector machine case-control study
Source: Mol Autism. 2016 Jan 19;7:5. doi: 10.1186/s13229-015-0067-3 (PMC4717545; doi:10.1186/s13229-015-0067-3)
Supplement: Additional file 2: — Document S2. Implementation details of the SVM-RFE procedure. (DOCX 286 kb) [file 13229_2015_67_MOESM2_ESM.docx]

**Additional details on the Support Vector Machine classification with Recursive Feature Elimination (SVM-RFE)**

The Support Vector Machine classification with Recursive Feature Elimination (SVM-RFE) was implemented in our analysis with two main goals: 1) to evaluate the predictive power of sMRI in Autism Spectrum Disorders (ASD); 2) to localize the subtle GM differences between subjects with ASD and controls. To achieve the first objective, an unbiased estimate of the SVM classification performance is obtained through the leave-pair-out cross validation procedure (LPO-CV). To achieve the second aim, we derived the so-called discrimination maps, which encode information about the GM areas more relevant for the classification, as identified in the SVM training procedure. The dimensionality reduction was implemented through the RFE procedure nested in the LPO-CV SVM classification. It consists in iteratively removing features (voxels) from the data set with the purpose of eliminating the major number of non-informative features/voxels while retaining the more discriminating ones. We reported in Figure S1 the trend of AUC as a function of the number of most discriminant retained voxels (*n_j_*) during the classification of the entire data sample, and of the subsamples of male and female subjects.


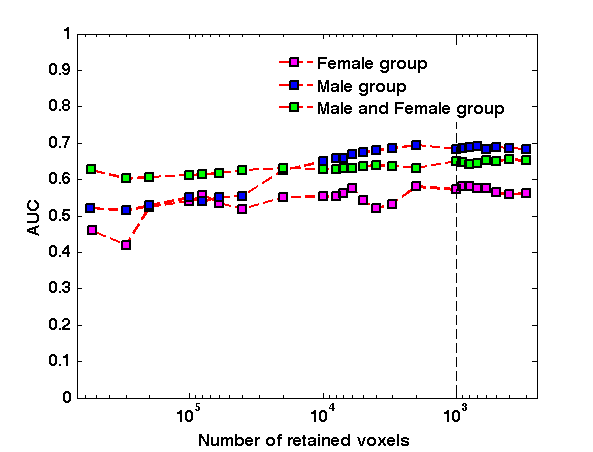


Figure S1. Classification performance measured in terms of AUC in LPO-CV for the male, female and entire samples of subjects. The values of AUC obtained with about 1000 retained voxels are: 0.68 for the male, 0.57 for the female subsets and 0.65 for the whole sample.

It can be noticed that only on the male group the RFE data reduction procedure leads to a sensible improvement of the classification performance, whereas for the female group and in particular for the whole data set there is not a clear trend of the curves. Similar trends have been reported on largely populated data samples, showing that not necessarily feature reduction steps lead to an improvement of the classification accuracy [s1-s3]. Nevertheless, in our analysis the SVM-RFE is a fundamental procedure to localize the neuroanatomical differences between two samples of subjects, leading us to the identification of an optimal set of thousand most discriminant voxels in the images.

To the choice of an optimal set of most discriminant voxels was suggested by the observation that the curves are particularly flat around one thousand voxels.

We reported in Figure S2 the discrimination maps obtained on the group of female subjects as a function of the number of retained voxels *n_j_*. Analogous maps are obtained on the group of male subjects and on the entire data sample. It can be noticed that as *n_j_* decreases the most important regions surviving the SVM-RFE become visible as small clusters of voxels.

*
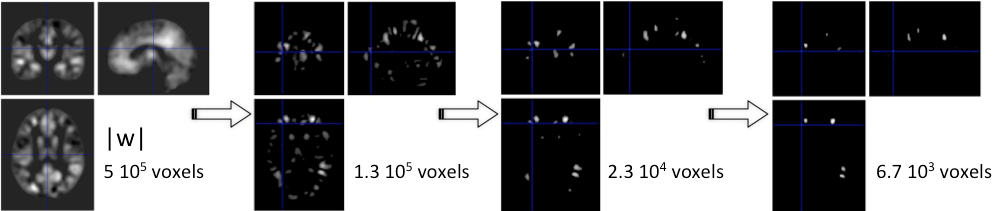
*

Figure S2. Discrimination maps shown at different steps during the SVM-RFE procedure for the subset of female subjects. It can be noticed that only the most informative voxels in the case-control binary discrimination problem survive the feature selection procedure.

For any iteration in the LPO-CV and for any value of *n_j_* a discrimination map is obtained on all data excluding one pair of matched subjects (for age and NVIQ). If the maps were inconsistent across the LPO-CV their average would provide a null/noise map. By contrast, as shown in Figure S3 for two different values of *n_j_*, we found out a strong similarity among the 38 maps for the female dataset, thus we averaged them and, to be conservative, we retained only the blobs common to all maps. Analogous considerations hold for the 38 and 76 maps for the male and the entire datasets, respectively.


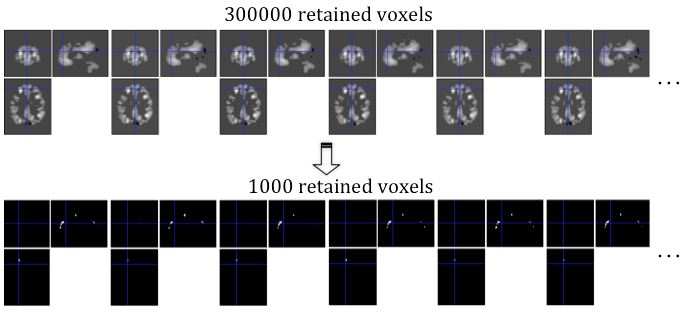


Figure S3. Consistency check across discrimination maps. Thirty-eight different discrimination maps are obtained in the LPO-CV procedure, training on all pairs of subjects except on one pair at each time, at each step of the RFE. The first six representative maps are shown as obtained training on 300000 (top row) and 1000 (bottom row) most discriminant voxels.

**References**

[s1] Cuingnet R, Gerardin E, Tessieras J, Auzias G, Lehéricy S, Habert MO *et al*; Alzheimer’s Disease Neuroimaging Initiative. **Automatic classification of patients with Alzheimer’s disease from structural MRI: a comparison of ten methods using the ADNI database**. *Neuroimage* 2011, **56**:766-781.

[s2] Chu C, Hsu AL, Chou KH, Bandettini P, Lin C; Alzheimer’s Disease Neuroimaging Initiative. **Does feature selection improve classification accuracy? Impact of sample size and feature selection on classification using anatomical magnetic resonance images.** *Neuroimage* 2012, **60**:59-70

[s2] Retico A, Bosco P, Cerello P, Fiorina E, Chincarini A, Fantacci ME. **Predictive Models Based on Support Vector Machines: Whole-Brain versus Regional Analysis of Structural MRI in the Alzheimer's Disease.** *J Neuroimaging* 2015, **25**:552-63.
